# An Anthocyanin-Rich Extract Obtained from Portuguese Blueberries Maintains Its Efficacy in Reducing Microglia-Driven Neuroinflammation after Simulated Digestion

**DOI:** 10.3390/nu12123670

**Published:** 2020-11-28

**Authors:** Diana Serra, Joana F. Henriques, Teresa Serra, Andreia Bento Silva, Maria Rosário Bronze, Teresa C. P. Dinis, Leonor M. Almeida

**Affiliations:** 1 CNC—Center for Neuroscience and Cell Biology, 3000-504 Coimbra, Portugal; joanafhenriques@gmail.com (J.F.H.); tcpdinis@ci.uc.pt (T.C.P.D.); malmeida@ci.uc.pt (L.M.A.); 2 Faculdade de Farmácia, Universidade de Coimbra, 3000-548 Coimbra, Portugal; 3 IBET—Instituto de Biologia Experimental e Tecnológica, 2780-157 Oeiras, Portugal; tserra@ibet.com; 4 ITQB—Instituto de Tecnologia Quimica e Biologica Antonio Xavier, Universidade Nova de Lisboa, 2780-157 Oeiras, Portugal; mbronze@ibet.pt; 5 Faculdade de Farmácia, Universidade de Lisboa, 1649-003 Lisboa, Portugal; abentosilva@ff.ulisboa.pt

**Keywords:** neuroinflammation, anthocyanins, phenolic acids, in vitro digestion, microglia, NF-kB, STAT1, ROS

## Abstract

Dietary polyphenols are multi-target compounds that have been considered promising candidates in strategies for the mitigation of neurological diseases, acting particularly through reduction of microglia-driven neuroinflammation. In this study, an anthocyanin-rich extract obtained from Portuguese blueberries was subjected to a simulated gastrointestinal digestion; after chemical characterisation, the potential of both non-digested and digested extracts to combat neuroinflammation was evaluated using a microglia N9 cell line. Although the extracts have markedly different chemical composition, both were efficient in reducing the production of either key inflammatory markers or reactive oxygen species and in enhancing reduced glutathione levels in activated cells. Furthermore, this protection was shown to be related to the suppression of nuclear factor kappa B (NF-kB) activation, and to a signal transducer and activator of transcription 1 (STAT1)-independent mechanism. These results demonstrate that the anthocyanin extract, after simulated digestion, maintains its efficacy against neuroinflammation, and can, therefore, assume a relevant role in prevention of neuroinflammation-related neurological disorders.

## 1. Introduction

Dietary polyphenols are a large family of chemically distinct natural compounds that have been shown to modulate various important cellular signalling pathways. They are of interest for the prevention and treatment of a number of chronic disorders [1]. In addition, their potential ability to modulate the microbiota-gut-brain axis makes these molecules promising candidates for application in neurological disorders [2]. 

Although the bioavailability of polyphenolic compounds remains controversial [3], there is agreement that polyphenols can achieve high concentrations in the intestinal tract [4]. Even though only limited data are available on actual concentrations achieved in the brain after oral consumption [5], several studies using animal models of brain diseases suggest many polyphenols have neuroprotective effects which have potential in the prevention and treatment of a number of brain disorders [6,7,8,9]. In a holistic perspective, considering the microbiota-gut-brain axis, despite their putative relatively low bioavailability, compounds able to target intestinal inflammation and dysbiosis may be of interest for neurological disorders, 

It is important to highlight and give due consideration to the fact that the bioavailability, and consequently, the biological activity, of dietary polyphenols depends on the chemical modifications that they undergo during the long journey in the gastrointestinal tract [10]. 

Recently, our research group has demonstrated that anthocyanins, a group of (poly)phenolic compounds predominant in red fruits, such as blueberries, can be used in a promising strategy to counteract gut inflammation in both cellular and animal models of disease [11,12,13]. In particular, Pereira et al. showed that an anthocyanin-rich extract, obtained from Portuguese blueberries, can reduce induced intestinal inflammation in mice even more efficiently than 5-aminosalicylic acid, a first-line drug in inflammatory bowel disease (IBD) [13]. From a molecular perspective, given that chronic intestinal inflammation may be intimately related to the development of neurological disorders, such as Alzheimer’s and Parkinson’s diseases, due to the well-recognised microbiota-gut-brain axis [14], it seemed pertinent to investigate whether this anthocyanin extract might reduce neuroinflammation. If the blueberry anthocyanins are shown to be successful in reducing both gut inflammation and neuroinflammation, they could be of interest to combat various neuroinflammation-related brain disorders. 

Neuroinflammation is a complex process that occurs in the brain, in which microglial cells play a relevant role [15]. These are resident innate immune cells. Among other important physiological functions, they are involved in brain surveillance and in immune defence. When activated, they change their morphology, migrate to the injured area, proliferate and produce several potent inflammatory and oxidant markers to re-establish the homeostasis of the central nervous system (CNS) [16]. However, in pathological conditions, activation of microglial cells becomes chronic and uncontrolled, and the subsequent effects can be noxious to the CNS, particularly by damaging neurons, oligodendrocytes or extracellular matrix structures [17]. These effects are not yet well understood mechanistically, but it is known that the overactivation of the NF-kB signalling cascade by microglial cells causes the overproduction of a number of inflammatory and oxidative markers, in particular nitric oxide (NO), prostaglandins (PGE) and cytokines, such as tumour necrosis factor-alpha (TNF-α) [18]. These contribute to the development of neurological disorders, namely Parkinson’s disease, Alzheimer’s disease, multiple sclerosis and others, such as Autism Spectrum Disorders [19,20,21]. 

Although some studies have already demonstrated that a number of polyphenolic compounds, including anthocyanins, can efficiently counteract neuroinflammation, the precise underlying molecular mechanisms have not been fully clarified [22,23,24].

The aim of this work was to study the ability of an anthocyanin-rich extract, obtained from Portuguese blueberries (*Vaccinium corymbosum* L.), to attenuate neuroinflammation. The underlying molecular mechanisms were analysed, using a N9 murine microglia cell line stimulated with a combination of lipopolysaccharide (LPS) and interferon (IFN) gamma, as a model of neuroinflammation. The anti-inflammatory and the antioxidant effects of the original extract (the anthocyanin-rich extract—ARE) were compared with those of a digested fraction (DIG), obtained after mimicking human digestion in vitro, to test to what extent the digestion-mediated chemical changes could interfere with such effects.

The comparison of anti-neuroinflammatory activity of both extracts was assessed not only for its ability to reduce the production or the expression of important inflammatory markers, namely NO, PGE2 and TNF-α, or inducible nitric oxide synthase (iNOS) and cyclooxygenase-2 (COX-2) enzymes, respectively, but also for its ability to reduce the release of reactive oxygen species (ROS) and upregulate the production of glutamate cysteine ligase (GCL) and of glutamate-cysteine ligase modifier subunit (GCLM), and consequently, the levels of reduced glutathione (GSH), in activated microglial cells. Furthermore, possible interferences with the activation of the nuclear factor kappa B (NF-kB) signalling pathway or with a Janus kinase/signal transducer and the activator of transcription-1 (JAK/STAT1) pathway, in activated microglial cells, were studied. 

## 2. Materials and Methods

### 2.1. Reagents 

General laboratory chemicals as well as 3-(4,5-dimethylthiazol-2-yl)-2,5-diphenyl tetrazolium bromide (MTT), dimethyl sulfoxide (DMSO), 2,7-dichlorofluorescein diacetate (DCFDA), lipopolysaccharide (LPS) (*Escherichia coli*, serotype 0111: B4), L-glutathione reduced form (GSH), o-phthalaldehyde (OPT), protease inhibitor cocktail, Folin-Ciocalteau reagent, pepsin from porcine gastric mucosa (P6887), pancreatin from porcine pancreas (P7545), bile extract porcine (B8631) and pefabloc (76,307) were purchased from Sigma Chemicals Co. (St. Louis, MO, USA). 

Cell culture reagents, namely, RPMI-1640 medium, foetal bovine serum (FBS) and phosphate-buffered saline (PBS) pH 7.4, were from Gibco-Invitrogen (Grand Island, NY, USA), except for penicillin and streptomycin, which were purchased from Sigma Chemicals Co. (St. Louis, MO, USA). IFN-γ was purchased from Invitrogen (Grand Island, NY, USA). For HPLC analyses, acetonitrile (Panreac, Barcelona, Spain) and formic acid (VWR-CHEM, Radnor, PA, USA) were used.

### 2.2. Plant Material 

Blueberries (*Vaccinium corymbosum* L., cultivar Bluecrop), from organic farms, were collected at the time of peak production in the central region of Portugal (Sever do Vouga, Aveiro, Portugal) and kept at −80 °C until use.

### 2.3. Preparation of the Anthocyanin-Rich Extract and Its Digested Fraction

The ARE was prepared as previously described [13]. The protocol was adapted according to Oszmianski et al. [25] and modified in accordance with Youdim et al. [26]. Briefly, a total extract was obtained from the homogenisation of 30 g of frozen fruits in 125 mL of methanol, acetone, water and formic acid mixture (40:40:20:0.1 *v*/*v*/*v*/*v*). The extract was then centrifuged at 2000× *g* for 10 min and the supernatant dried by rotatory evaporation, at 40 °C, under vacuum. The resulting residue was dissolved in deionised water and applied to an activated Sep-Pak C18 column (Waters Corporation, Milford, MA, USA). This column was then washed with two volumes of acidified water (0.01% HCl) to remove sugars and phenolic acids, and two volumes of ethyl acetate to elute other phenolic compounds. The anthocyanins were eluted only in the presence of acidified methanol (0.01% HCl). This fraction was then dried at a low pressure and then resolubilised in 3 mL of 0.9% NaCl. The anthocyanin-rich fraction was kept at −80 °C, under nitrogen, until use. 

The ARE was further subjected to conditions that mimicked gastrointestinal digestion using the standardised static in vitro digestion method suitable for food, as described by Minekus et al. [27]. A part of the ARE was diluted 2.5 times (to a final concentration of 3.6 mg/mL in terms of total phenolic contents) and further subjected to conditions that mimicked gastrointestinal digestion. For this, the anthocyanin-rich fraction (5 mL) was first mixed with a simulated salivary fluid (5 mL), which constituted 15.09 mM KCl, 1.35 mM KH_2_PO_4_, 13.68 mM NaHCO_3_, 0.15 mM MgCl_2_(H_2_O)_6_, 0.06 mM NH_4_(CO_3_)_2_, 1.5 mM CaCl_2_(H_2_O)_2_ and 1.1 mM HCl, and was incubated for 2 min at 37 °C, under agitation. The mixture was then subjected to a simulated gastric fluid (10 mL), which constituted 6.9 mM KCl, 0.9 mM KH_2_PO_4_, 25 mM NaHCO_3_, 47.2 mM NaCl, 0.12 mM MgCl_2_(H_2_O)_6_, 0.5 mM NH_4_(CO_3_)_2_, 0.3 mM CaCl_2_(H_2_O)_2_, 15.6 mM HCl and pepsin (2000 U/mL), and after a pH adjustment to 3, the mixture was incubated at 37 °C, and shaken at 100 rpm for 2 h. Subsequently, intestinal conditions were mimicked, with the mixture subjected to a simulated intestinal fluid (20 mL) that constituted 6.8 mM KCl, 0.8 mM KH_2_PO_4_, 85 mM NaHCO_3_, 38.4 mM NaCl, 0.33 mM MgCl_2_(H_2_O)_6_, 0.6 mM CaCl_2_(H_2_O)_2_, 8.4 mM HCl, pancreatin (200 U/mL) and bile salts (160 mM); after adjustment to pH 7, the mixture was incubated at 37 °C, with shaking at 100 rpm, for 2 h. After full digestion, the reaction was stopped with pefabloc (5 mM); the mixture was then centrifuged for 40 min, at 10,000× *g*, and kept at −20 °C. Then, the mixture was acidified with 10% (*v*/*v*) formic acid and centrifuged at 2500× *g*, for 10 min. The soluble material was passed through a Sep-Pak C18 column (Waters Corporation, Milford, MA, USA) to separate the phenolic compounds from bile salts [28]. C18 solid phase columns were pre-equilibrated in deionised water acidified with 0.25% (*v*/*v*) formic acid and then washed with 0.25% (*v*/*v*) formic acid in 25% (*v*/*v*) acetonitrile. Then, this extract was dried in a rotary evaporator at low pressure, and the residue was resolubilised in 2.5 mL of sterile deionised water. The aliquots were kept at −20 °C until use. 

### 2.4. Quantification of Total Phenolic Content

The quantification of total phenolic content was performed by the Folin-Ciocalteau method, as described by Georgé et al. [29]. Results were expressed as milligrams of gallic acid equivalents (GAE) per volume of ARE (L).

### 2.5. Determination of the Phenolic Profile by HPLC-DAD-MS/MS

The HPLC analyses were performed on a Waters Alliance 2695 (Waters^®^, Ireland) equipped with a quaternary pump, solvent degasser, auto sampler and column oven, coupled to a Photodiode Array Detector Waters 996 PDA (Waters^®^, Ireland). 

Separation was performed on a reversed-phase column (SunFire^®^ C18, 150 × 4.6 mm; 3.5 µm; Waters^®^), at 35 °C, using an injection volume of 20 µL. The mobile phase consisted of Milli-Q water containing 0.5% (*v*/*v*) formic acid (eluent A) and acetonitrile (eluent B), at a flow rate of 0.30 mL/min. The following gradient program was used: 0–8 min at 95% A; 8–9 min from 95 to 90% A; 9–54 min from 90 to 85% A; 54–94 min from 85 to 45% A; 94–96 min from 45 to 10% A; 96–100 min at 10% A, finally returning to the initial conditions for 20 min. A photodiode array detector was used to scan the wavelength absorption from 200 to 700 nm. 

Tandem mass spectrometry (MS/MS) detection was performed on a Micromass^®^ Quattro Micro triple quadrupole (Waters^®^, Ireland), using an electrospray ionisation (ESI) source operating at 120 °C and applying a capillary voltage of 2.5 kV and source voltage of 30 V. The compounds were ionised in positive and negative ion modes, and the spectra of the column eluate were recorded in the range of 60–1100 m/z. MassLynx software (version 4.1) was used to control the system, for data acquisition and processing. High purity nitrogen (N_2_) was used as drying gas and as a nebulising gas. Ultra-high purity argon (Ar) was used as collision gas. 

### 2.6. Cell Culture 

N9 cells (immortalised mouse microglial cells) were kindly provided by Professor Conceição Pedroso Lima (CNC—Center for Neuroscience and Cell Biology, University of Coimbra, Coimbra, Portugal). Cells were grown in RPMI supplemented with 5% FBS and 1% penicillin/streptomycin at 37 °C in a humidified atmosphere of 5% CO_2_. Cells were sub-cultured at sub-confluenced (60–80%) with trypsin-EDTA and used between the 19th and the 28th passage. Cells were seeded in 12-well plates (8 × 10^4^ cells/well) or in 6-well plates (2.4 × 10^5^ cells/well), depending on the assays, and treated according to the various experimental purposes required.

Cells were pre-treated with the ARE or DIG, for 3 h, before the exposure to the stimulus, 1 µg/mL LPS and 0.6 ng/mL IFN-γ, and then maintained with the inflammatory stimulus for different time intervals, depending on the assay.

### 2.7. Cell Viability 

Cell viability was assessed by the mitochondrial-dependent reduction of 3-(4,5-dimethylthiazol-2yl)-2,5-diphenyltetrazolium bromide (MTT) to formazan, which is directly proportional to the number of living cells. After the incubation of cells with ARE or DIG, 8 × 10^4^ cells/well in a 12-wellplate, for 24 h, the culture medium was removed, the cells were washed with PBS and incubated with MTT (0.5 mg/mL) for 1 h, at 37 °C. Then, the supernatant was removed, and the formazan crystals were dissolved in DMSO (450 µL). The extent of formazan formation was recorded at 530 nm in a plate reader (Bio-TEK Synergy HT, Izasa S.A., Madrid, Spain). Results were expressed as a percentage of control cells, i.e., non-treated cells.

### 2.8. Measurement of Nitric Oxide Production

NO production was determined by measuring the amount of nitrite accumulated in cell culture supernatants, by using the Griess method. Briefly, N9 cells were treated as above and after 24 h, extracellular media was mixed with Griess reagent for 10 min, at room temperature. The absorbance was read at 540 nm using a plate reader (Bio-TEK Synergy HT, Izasa S.A., Madrid, Spain). The values were reported relative to protein content, as measured by the Pierce BCA protein assay kit, according to the manufacturer’s specifications (Thermo Fisher Scientific, Waltham, MA, USA). Results were expressed as a percentage of stimulated cells, i.e., LPS- and IFN-γ-treated cells.

### 2.9. Assessment of Prostaglandin E_2_ Production

N9 cells were pre-incubated with the polyphenolic extracts under study and then treated with LPS and IFN-γ, as described above. After 16 h of stimulation, supernatants were collected and processed for PGE_2_ quantification, by using a competitive immunoassay kit (PGE_2_ EIA Kit, from Enzo Life Science, Farmingdale, New York, USA), according to the manufacturer’s instructions. The results were normalised in terms of protein content, as measured by the Pierce BCA protein assay kit, according to the manufacturer’s specifications (Thermo Fisher Scientific, Waltham, MA, USA). Results were expressed as a percentage of stimulated cells, i.e., LPS- and IFN-γ-treated cells.

### 2.10. Quantitative Real-Time RT-PCR (qRT-PCR) for Evaluation of iNOS, COX-2, GCLC and GCLM mRNA Production

Total RNA was extracted from N9 cells seeded in six-well-plates (2.4 × 10^5^ cells/well) by using the RNA extraction kit Aurum^TM^ Total RNA Mini (Bio-Rad, Hercules, CA, USA), according to the manufacturer’s instructions and as previously described. 

The primers for iNOS, COX-2, GCLC, GCLM and the housekeeping gene HPRT-1 (hypoxanthine phosphoribosyltransferase-1) were designed using the Beacon Designer software (PREMIER Biosoft International, Palo Alto, CA, USA), and the primers′ sequences were: iNOS, sense 5′-ATACAAGATGACCCTAAGA-3′, antisense 5′-GGATTCTGGAACATTCTG-3′; COX-2, sense 5′-ATCAGACCTTCCTTGTAT-3′; antisense 5’-CACACTCATAGTTAAGACA-3′; GCLC, sense 5′-ACTTCCTTCTACATACAC-3′; antisense 5′-GCACTGAGTTGATTATTC-3′ GCLM, sense 5′-TAGCAGTCTACCAGTAAT-3′; antisense 5′-AGATAAGAGGTGGAAGAA-3′ and HPRT-1, sense 5′-CCATTCCTATGACTGTAGA-3′, antisense 5′-CTTCAACAATCAAGACATTC-3′. 

The efficiency of the amplification reaction for each gene was calculated by running a standard curve of serially diluted cDNA sample. Gene expression was analysed using the Bio-Rad CFX Manager 3.0 software (Bio-Rad, Hercules, CA, USA), which enables the analysis of the results with the Pfaffl method. The results for each gene of interest were normalised against HPRT-1, the housekeeping gene, found to be stable under our experimental conditions and expressed as a percentage of stimulated cells, in the case of iNOS and COX-2 and as a percentage of negative control cells, i.e., non-stimulated cells, in the case of GCLC and GCLM subunits.

### 2.11. Assessment of TNF-Alpha Production

N9 cells were pre-incubated with the polyphenolic fractions and then treated with LPS and IFN-γ, as described above. After 16 h of stimulation, supernatants were collected and processed for TNF-α quantification, by using an in vitro enzyme-linked immunosorbent assay from Raybiotech (Biocompare, Norcross, Georgia, USA), according to the manufacturer’s instructions. The values were reported, relative to the protein content, as measured by the Pierce BCA protein assay kit, according to the manufacturer’s specifications (Thermo Fisher Scientific, Waltham, MA, USA). Results were expressed as a percentage of stimulated cells, i.e., LPS- and IFN-γ-treated cells.

### 2.12. Evaluation of Intracellular Reactive Oxygen Species Production

Intracellular ROS were assessed by using the non-fluorescent probe 2′, 7′-dichlorodihydrofluorescein diacetate (DCFH_2_-DA), which permeates cell membranes and may be oxidised by reactive species, yielding the fluorescent 2′, 7′-dichlorofluorescein (DCF). Briefly, cells in 12-well plates (8 × 10^4^ cells/well) were previously pre-incubated with the polyphenolic extract under study and further subjected to the combination of LPS and IFN-γ for 24 h. After that, the cells were incubated with 25 μM DCFH_2_-DA in PBS, at 37 °C, in the dark for 30 min. Cells were then washed with PBS and maintained in 0.5 mL of PBS during the observation in an inverted fluorescence microscope (Zeiss Axiovert 40), using an FITC filter. The mean fluorescence intensity was analysed using ImageJ software (National Institute of Mental Health, Bethesda, Maryland, USA). Results were expressed as a percentage of stimulated cells, i.e., LPS- and IFN-γ-treated cells.

### 2.13. Evaluation of GSH

The intracellular content of reduced glutathione (GSH) was determined by a fluorimetric assay, as described by Hissin and Hilf [30]. Briefly, GSH was measured upon its reaction with a fluorescent reagent, o-phthalaldehyde (OPT), at pH 8. After 24 h of incubation, cells were washed twice with cold PBS, detached and resuspended in 250 µL of 100 mM Na_2_HPO_4_, pH 8. After the addition of an equal volume of 0.6 M HClO_4_, the mixture was maintained on ice for 5 min. After vigorous vortexing, the cellular extracts were centrifuged at 14,000 rpm for 5 min, at 4 °C. Supernatants were collected and the respective pellets were resuspended in 1 M NaOH for protein quantification. For GSH quantification, 100 µL of each sample was added to 1800 µL of 100 mM Na_2_HPO_4_ buffer pH 8, and to 100 µL of OPT and maintained in the dark, at room temperature, for 15 min, before fluorescence detection. A standard curve was also prepared with known concentrations of GSH. Fluorescence intensity was read in a plate reader (Bio-TEK Synergy HT, Izasa S.A., Madrid, Spain) (excitation and emission wavelengths at 350 and 420nm, respectively). Cellular GSH contents were calculated using concurrently run standard curves and expressed as nmol GSH per milligram of protein. Cellular protein was determined by using the Pierce BCA protein assay reagent, according to the manufacturer’s specifications (Thermo Fisher Scientific, Waltham, MA, USA). Results were expressed as a percentage of stimulated cells, i.e., LPS- and IFN-γ-treated cells.

### 2.14. Evaluation of NF-kB (p65) Activity

The DNA-binding activity of NF-kB-p65 was measured in nuclear extracts using the TransAM^TM^ NF-kB-p65 protein assay (Active Motif, Carlsbad, CA, USA), an ELISA-based method with high sensitivity and reproducibility. 

For preparation of nuclear extracts, washed cells were lysed in an ice-cold buffer containing 10 mM Tris–HCl, 10 mM NaCl, 3 mM MgCl_2_, 0.5% Nonidet P-40 and 1% protease inhibitor cocktail, pH 7.5, for 5 min on ice. Afterwards, lysates were centrifuged at 5000 rpm for 5 min at 4 °C and the supernatants (cytoplasmic extracts) were collected and stored at −20 °C. The pellets were collected and resuspended in 30 µL of Complete Lysis Buffer (a solution provided by Active Motif, Carlsbad, CA, USA) and left on ice for 30 min. Then, lysates were centrifuged at 14,000 rpm, for 10 min at 4 °C, and the supernatants (nuclear extracts) were saved at −80 °C. 

DNA binding activity of p65 was evaluated in 20 µg of nuclear protein, according to the manufacturer’s protocol, and the results were expressed in relative terms, as a percentage of stimulated cells, i.e., LPS- and IFN-γ-treated cells.

### 2.15. Evaluation of p-STAT1 (Tyr701) Levels

The quantification of pSTAT1 (Tyr701) levels in cell lysates was performed by using an in vitro enzyme-linked immunosorbent assay (Abcam, Cambridge, UK), an ELISA-based method with high sensitivity and reproducibility. 

For preparation of nuclear extracts, washed cells were lysed in an ice-cold buffer containing 10 mM Tris–HCl, 10 mM NaCl, 3 mM MgCl_2_, 0.5% Nonidet P-40 and 1% protease inhibitor cocktail, pH 7.5, for 5 min on ice. Afterwards, lysates were centrifuged at 5000 rpm for 5 min at 4 °C and the supernatants (cytoplasmic extracts) were collected and stored at −20 °C. The pellets were collected and resuspended in 30 µL cell lysis buffer, provided by Abcam (Cambridge, UK), supplemented with a protease inhibitor cocktail and left on ice for 30 min. Afterwards, lysates were centrifuged at 14,000 rpm, for 10 min at 4 °C, and the supernatants were collected and stored at −80 °C. 

The levels of pSTAT-1 (Tyr701) were measured according to the manufacturer’s protocol. Results were expressed as a percentage of the control cells, i.e., non-treated cells.

### 2.16. Statistical Analysis 

All data were expressed as means ± SEM of at least three independent assays, each one in duplicate. Differences between groups were analysed by one-way analysis of variance (ANOVA), Tukey’s test was used as appropriate. Values of *p* < 0.05 were accepted as statistically significant.

## 3. Results 

### 3.1. Chemical Characterisation of the Anthocyanin-Rich Extract Obtained from Portuguese Blueberries and of Its Digested Fraction

The total phenolic contents of the ARE and of the DIG were 9000 mg/L and 850 mg/L, respectively, in terms of gallic acid equivalents. 

**Figure 1:**
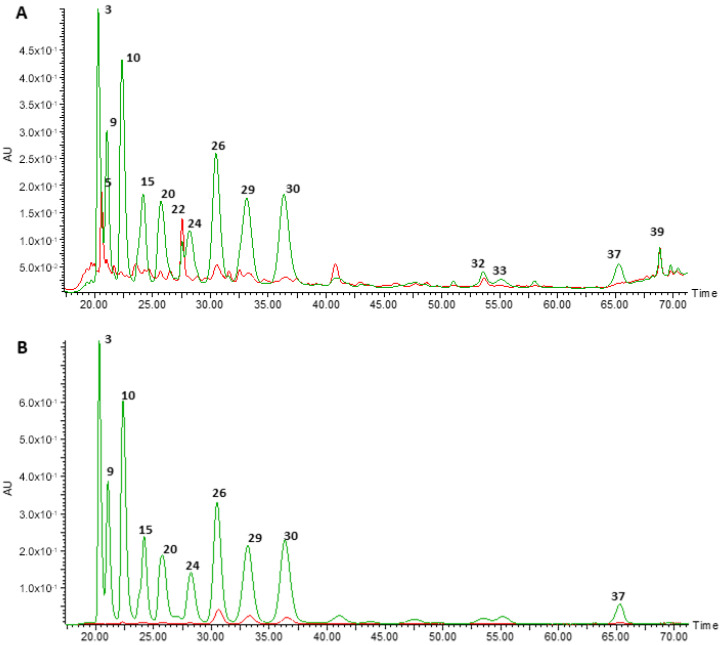
Comparison of the chromatographic profiles (from 17 to 72 min) of the original extract of Portuguese blueberries—ARE (green)—and the digested extract—DIG (red)—extracts at (**A**) 280 and (**B**) 525 nm. Peaks are labelled as described in Table 1.

To characterise the phenolic composition of the ARE and the DIG, both were analysed by HPLC-DAD-MS/MS. Figure 1 shows the chromatographic profiles of the ARE (green) and DIG (red) detected at 280 and 525 nm, characteristic wavelengths of phenolic compounds and of anthocyanins, respectively. The putatively identified compounds detected are given in Table 1.

As previously reported [13], the ARE showed a wide variety of anthocyanin molecules, in particular, malvidin, cyanidin, delphinidin, petunidin and peonidin, conjugated with either galactose, glucose or arabinose. The main molecular species detected, according to peak areas, are malvidin 3-galactoside, malvidin 3-arabinoside and malvidin 3-glucoside (Figure 1 and Table 1). During the in vitro digestion, a significant decrease in concentration of most of the anthocyanins was detected, notably cyanidin, delphinidin and petunidin (Table 1). However, malvidin conjugates remained in the extract even after simulated digestion (Table 1). In contrast, other phenolic compounds increased after digestion (Table 1), in particular phenolic acids (such as protocatechuic acid 4-*O*-glucoside, ferulic acid hexoside, dicaffeoylquinic acid, caffeoyl quinic acid and ellagic acid 4-acetylpentoside) and other flavonoids (such as resveratrol, myricetin and quercetin derivatives).

### 3.2. Neither the Original Anthocyanin Extract Nor Its Digested Fraction, at the Concentration Used, Affected the Viability of N9 MICROGLIAL Cells

To evaluate the cytotoxic effect of the ARE and of the DIG, the MTT assay was performed following 24 h of cell incubation. The ARE extract, in the concentration range between 2.5 µg/mL and 10 µg/mL, did not affect the viability of N9 cells. However, a small decrease in the cell viability was observed when used at a concentration of 20 µg/mL (supplementary Figure S1). The DIG, in the concentration range (2.5–20 µg/mL), did not affect the percentage of the viability of microglial cells when compared to the control cells (cells not treated). Thus, the concentration of 10 µg/mL was selected for both phenolic fractions to perform the next experiments. 

Note that the combination of LPS and IFN-γ, under the experimental conditions used as a cell stimulus, promoted a 15% decrease in the microglial cell viability (data not shown). 

### 3.3. Both the Original Anthocyanin Extract and Its Digested Fraction Reduced the Secretion of NO and PGE2, by Downregulating the mRNA Production of iNOS and COX-2, in Stimulated N9 Microglial Cells

In order to assess the ability of the ARE to inhibit the production of two key pro-inflammatory mediators, the NO and the PGE2, generated by LPS- and IFN-γ-stimulated N9 cells, and to compare the anti-inflammatory effect of the ARE with that of the DIG, the levels of these markers were monitored. 

**Figure 2:**
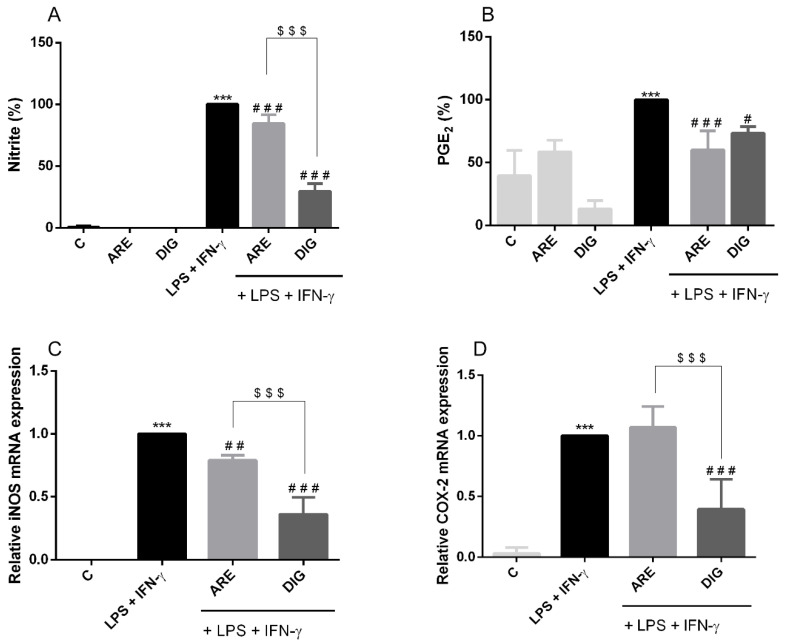
The original extract of Portuguese blueberries (ARE) and the digested extract (DIG) inhibit the production of key pro-inflammatory mediators induced by lipopolysaccharide (LPS) and interferon-gamma (IFN-γ), in N9 microglial cells. Cells were pre-incubated with the same concentration of total polyphenols (10 µg/mL) provided from ARE or DIG for 3 h, and then stimulated with LPS and IFN-γ for a certain period of time. NO (**A**) and PGE_2_ (**B**) production or expression of iNOS (**C**) and COX-2 (**D**) enzymes by microglial cells were measured as described in the “Materials and Methods” and expressed as a percentage relative to the activated cells (black bars). Values are mean ± SEM of at least three different experiments, in duplicate. *** *p* < 0.001 vs. negative control (**C**, non-stimulated cells), ^#^ *p* < 0.05, ^##^ *p* < 0.01, ^###^ *p* < 0.001 vs. positive control (LPS + IFN-γ stimulated cells) and ^$$$^ *p* < 0.001 vs. ARE plus LPS and IFN-γ.

As can be seen in Figure 2A, the combination of LPS and IFN-γ produced a significant increase in NO levels, measured in terms of cellular nitrite formation, compared with control cells (non-treated cells). As also illustrated in Figure 2A, treatment of cells with 10 µg/mL of phenols of ARE or DIG, for 3 h before LPS/IFN-γ stimulation, reduced the nitrite levels significantly (about 80% and 30%, respectively), indicating a greater efficiency of the digested fraction when compared to the original form of the anthocyanin-rich extract. 

From Figure 2B, it is clear that the pro-inflammatory stimulus (LPS and IFN-γ) was able to increase the PGE2 levels in N9 cells and that the pre-incubation of these cells with the ARE or the DIG led to a significant decrease in the production of this important inflammatory marker in activated microglial cells. Although the effect of the DIG in PGE2 levels was not as marked as that obtained with the ARE, in vitro digestion did not have a negative impact on the anti-inflammatory efficiency of the DIG when compared to the ARE. 

To further evaluate whether the reduction of NO and PGE2 production was directly related to the downregulation of the expression of iNOS and COX-2, the mRNA production of these two enzymes was determined by qRT-PCR. Comparing Figure 2C,D, we can see that the levels of mRNA of both enzymes are barely detectable in control cells but are strongly induced in cells stimulated with LPS and IFN-γ. In particular, the DIG is seen to be highly efficient in reducing the production of iNOS and COX-2 mRNA in stimulated cells and is even more efficient than the ARE. We can, therefore, conclude that the in vitro digestion improves the anti-inflammatory effect of the ARE.

### 3.4. The Anthocyanin-Rich Extract and Its Digested Fraction Reduced the Production of TNF-α in Stimulated N9 Microglial Cells

The ability of the extracts to reduce the production of an important inflammatory cytokine, the TNF-α, by N9 cells stimulated with LPS and IFN-γ, was measured to complement the previous results in terms of evaluation of the anti-inflammatory potential of ARE and DIG extracts.

**Figure 3:**
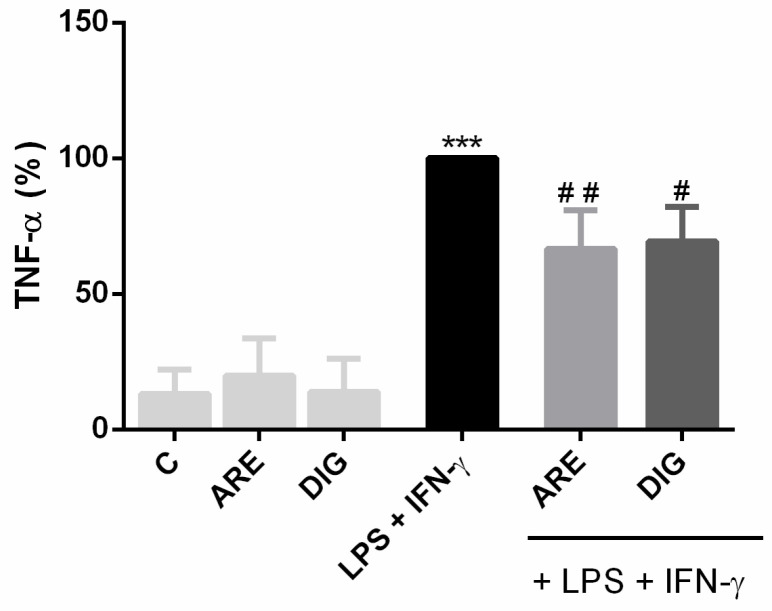
Both the original extract of Portuguese blueberries (ARE) and the digested extract (DIG) inhibit TNF-α production, in LPS- and IFN-γ-exposed N9 cells. Cells were pre-incubated with the same concentration of total polyphenols (10 µg/mL) provided for ARE or DIG for 3 h, and then stimulated with LPS and IFN-γ for 16 h. TNF-α production was evaluated as described in the “Materials and Methods” and expressed as a percentage relative to the activated cells (black bar). Values are mean ± SEM of at least three different experiments, in duplicate. *** *p* < 0.001 vs. negative control (C, non-stimulated cells), ^#^ *p* < 0.05 and ^##^ *p* < 0.01 vs. positive control (LPS + IFN-γ stimulated cells).

As can be seen in Figure 3, the combination of LPS and IFN-γ significantly enhanced the production of TNF-α by N9 microglial cells, while pre-treatment of the cells, with either the ARE or the DIG, was efficient in significantly reducing the production of this pro-inflammatory cytokine. These results also clearly demonstrate that the ability of ARE to reduce TNF-α production in activated N9 cells is maintained, even after its in vitro digestion. 

### 3.5. The Anthocyanin-Rich Extract and Its Digested Fraction Significantly Decreased the Intracellular Production of ROS in Stimulated N9 Microglial Cells

Since the inflammatory and oxidative processes are closely related phenomena, mechanistically, it seemed pertinent to check whether the ARE and the DIG could prevent the intracellular generation of reactive oxygen species by activated N9 microglial cells. 

**Figure 4:**
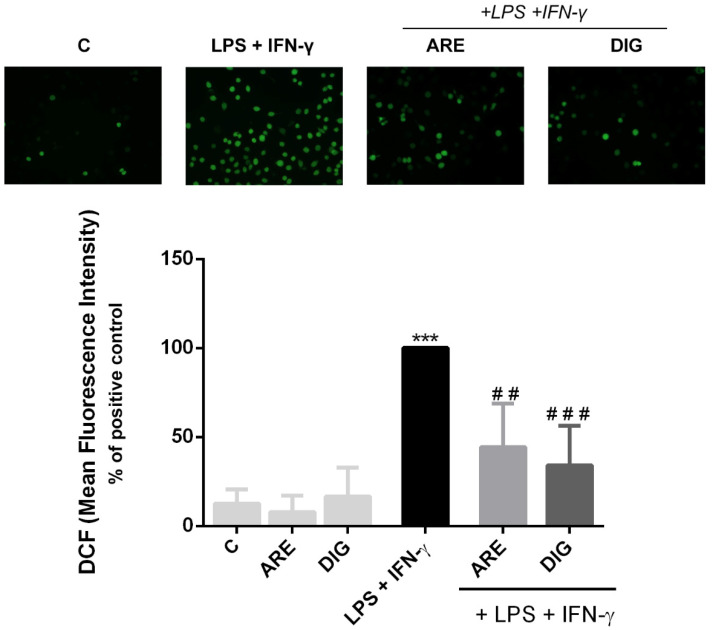
The original extract of Portuguese blueberries (ARE) and the digested extract (DIG) significantly reduce ROS production in LPS- and IFN-γ-exposed N9 cells. Cells were pre-incubated with the same concentration of total polyphenols (10 µg/mL) provided from ARE or DIG for 3 h, and then stimulated with LPS and IFN-γ for 24 h. Reactive oxygen species (ROS) production was evaluated by oxidation of the probe dichlorohydrofluorescein, as described in the “Materials and Methods”, and expressed in terms of percentage of fluorescence intensity relatively to positive control (LPS + IFN-γ stimulated cells) (black bar). Representative images obtained by fluorescence microscopy (400×) of cells, at 24 h after LPS + IFN-γ treatment in the absence or presence of ARE or DIG, are presented at the top. Values are mean ± SEM of at least three different experiments, in duplicate. *** *p* < 0.001 vs. negative control (C), ^##^ *p* < 0.01 and ^###^ *p* < 0.001 vs. positive control (LPS + IFN-γ stimulated cells).

As can be seen in Figure 4, the production of reactive oxygen species by N9 cells was significantly enhanced after 24 h exposure to the mixture of LPS and IFN-γ. Both the bar graph of Figure 4 and the pictures above the graph show that the ARE and the DIG were able to significantly decrease the ROS levels produced by activated microglial cells.

### 3.6. The Anthocyanin-Rich Extract Subjected or Not to In Vitro Digestion Significantly Increased the Levels of Reduced Glutathione (GSH), by Enhancing the mRNA Production of GCLC and GCLM, in Activated N9 Microglial Cells

To obtain more details on the antioxidant potential of the ARE and its DIG fraction, the levels of intracellular reduced glutathione (GSH) in microglial cells were monitored. GSH is the main low-molecular-weight antioxidant in the brain, and has an important role in the maintenance of redox homeostasis in microglial cells [27].

Glutamate cysteine ligase (GCL) is the enzyme responsible for the first-limiting step of the production of GSH in cells. Because of this, the production of GCL was evaluated at a transcriptional level by qRT-PCR. GCL consists of catalytic (GCLC) and modifier (GCLM) subunits. 

**Figure 5:**
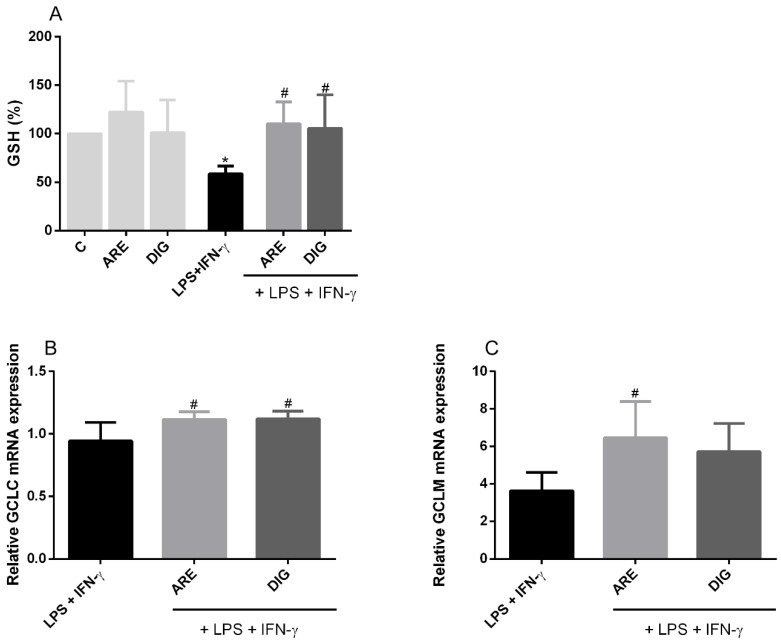
The original extract of Portuguese blueberries (ARE) and the digested fraction (DIG) reduce GSH production by downregulating the mRNA expression of GCL, in LPS- and IFN-γ-stimulated N9 cells. Cells were pre-incubated with the same concentration of total polyphenols (10 µg/mL) provided from ARE or DIG for 3 h, and then stimulated with LPS and IFN-γ for a certain time. GSH (**A**), GCLC (**B**) and GCLM (**C**) production was evaluated as described in the “Materials and Methods”. GSH production was expressed as a percentage relative to the activated cells (dark bars) and GCLC and GCLM mRNA production was expressed as a percentage relative to the control cells (light grey bars). Values are mean ± SEM of at least three different experiments, in duplicate. * *p* < 0.05 vs. Control and ^#^ *p* < 0.05 vs. LPS and IFN-γ.

The combination of LPS and IFN-γ substantially reduced the content of this key intracellular antioxidant in N9 cells, after 24 h of exposure to the stimulus, as can be seen in Figure 5A. However, GSH depletion was prevented if the microglial cells were pretreated with the ARE or the DIG. This is in agreement with results depicted in Figure 5B,C, where both GCLC and GCLM mRNA levels were greatly increased in cells pre-treated with the DIG or with the ARE. 

### 3.7. Both the Original Anthocyanin-Rich Extract and the Digested Fraction Reduced NF-kB-p65 Activation without Interfering with JAK/STAT1 Pathway, in LPS- and IFN-γ-Exposed N9 Cells

Since NF-kB activation is critical for the development of the inflammatory and oxidative processes in microglial cells [14], it seemed relevant to check whether the anti-inflammatory and antioxidant protection afforded by both the ARE and DIG were related to the attenuation of the activation of this important transcriptional factor.

**Figure 6:**
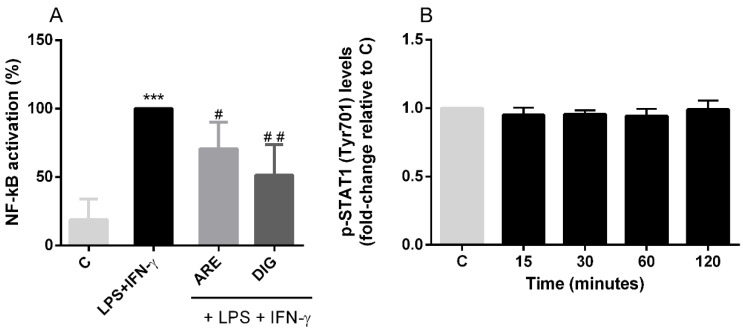
The original extract of Portuguese blueberries (ARE) and the digested fraction (DIG) attenuate NF-kB activation but not interfere with STAT1 activation, in LPS- and IFN-γ-exposed N9 cells. (**A**) Cells were pre-incubated with the same concentration of total polyphenols (10 µg/mL) provided from ARE or DIG for 3 h, and then stimulated with LPS and IFN-γ for 2 h. NF-kB activation was evaluated as described in the “Materials and Methods” and expressed as a percentage relative to the activated cells (dark bars). (**B**) Cells were incubated with LPS and IFN-γ for 15 min, 30 min, 1 h and 2 h. STAT1 activation was evaluated as described in the “Materials and Methods” and expressed as a percentage relative to the control cells (non-stimulated cells). Values are mean ± SEM of at least three different experiments, in duplicate. *** *p* < 0.001 vs. Control and ^#^ *p* < 0.05 and ^##^ *p* < 0.01 vs. LPS and IFN-γ.

Initially, a time-course study was performed, and the maximum activation of NF-kB-p65 was obtained after 2 h of N9 cells exposure to LPS and IFN-γ (data not shown). At this reaction time, under our assay conditions, both the ARE and the DIG could significantly reduce the activation of NF-kB-p65, as shown in Figure 6A.

Since the IFN-γ response has, classically, been related to the activation of JAK/STAT signalling, and it is commonly thought that, after IFN-γ activation, the levels of phosphorylated STAT1 increases in the cell nucleus, promoting gene transcription, it seemed appropriate to study whether the protection afforded by the polyphenolic compounds was related to the STAT1 signalling cascade in microglial cells. Surprisingly, under our experimental conditions, LPS and IFN-γ did not alter the levels of p-STAT1 (Tyr701) in the nucleus of microglial cells compared to the control, non-stimulated cells (Figure 6B). Therefore, the protection afforded by the polyphenolic compounds under study was not related to the STAT1 signalling cascade in LSP- and IFN-γ-stimulated-microglial cells.

## 4. Discussion

Polyphenols are naturally occurring compounds, abundant in conventional Western diets, that can achieve high concentrations at the intestinal compartment after oral consumption, and, consequently, have been considered as interesting candidates for the prevention and treatment of gut disorders, particularly those with a major inflammatory and oxidant basis [4].

The impact of chronic intestinal inflammation and dysbiosis on the development of neurological disorders has aroused great interest in recent years [14]. Because of the limited efficiency and the severe adverse effects of current treatment options for neurological disorders, the search for alternative, safer and multitarget therapies is of considerable importance to the scientific community. In this context, compounds that combine the capacity to counteract intestinal inflammation and modulate gut microbiota composition with the ability to counteract neuroinflammation, thus contributing to neuroprotection, may be doubly valuable in the prevention and treatment of many neurological disorders [2,37]. Previous in vitro and in vivo research from our group, and others, have shown that anthocyanins, a specific group of polyphenols, can be beneficial for the prevention and treatment of chronic intestinal inflammation [11,13,38].

Some studies have demonstrated that when anthocyanins, such as malvidin-3-glucoside and cyanidin-3-glucoside, modulate gut microbiota composition, they enhance the growth of beneficial bacteria, such as *Bifidobacterium* spp. and *Lactobacillus* spp. [39,40]. Although the potential of anthocyanins to counteract neuroinflammation has already been investigated [41,42,43], there is still much to be understood mechanistically.

In addition, anthocyanins may suffer several chemical alterations after oral consumption, caused by the digestion process, which can influence their bioactivity, and the effect of this must be considered [44]. 

We report a study of the main chemical alterations that occur in anthocyanins of an anthocyanin-rich extract, obtained from Portuguese blueberries, after simulated digestion, and subsequently evaluate and compare the efficiency of the DIG with that of the ARE in counteracting the neuroinflammatory process, using a microglial cell line. We note that in vitro simulated gastrointestinal digestion has been extensively used to evaluate the bioavailability of different polyphenols, since it is reproducible, time saving and low cost [35,45,46].

Following simulated digestion under our experimental conditions, loss of most of the anthocyanins was observed in the ARE. Changes in the composition of an original polyphenolic extract on simulated digestion have been previously described by other authors, and may be due to factors such as degradation or alteration of the initial chemical structure caused by binding to proteins and pH changes [35]. Significant decreases in the amounts of anthocyanins have been reported after pancreatic/small intestine digestion, and explained, at least in part, by their transformation to colourless chalcones and subsequent ring cleavage at alkaline pH. These processes can affect their bioavailability and their identification in the DIG by HPLC-DAD [47]. A number of studies have corroborated this hypothesis [48,49], and we cannot exclude this possibility to explain, in part, the observed decrease of anthocyanin amounts in the DIG. 

However, the present results show the higher stability of malvidin derivatives, as compared to cyanidin and delphinidin derivatives, to the degradation promoted by simulated digestion, and are in agreement with the suggestion that having more hydroxyl groups in the anthocyanins’ B-ring structure increases susceptibility to such digestive processes [50]. Malvidin derivatives, with fewer hydroxyl groups than cyanidin and delphinidin ones, are, thus, more resistant to the digestion degradation.

As previously reported, Portuguese blueberries contain a great variety of monoglycosides (glucoside, galactoside and arabinoside) of cyanidin, malvidin, delphinidin, petunidin and peonidin [13], which are excreted unmetabolised by humans and rats [51]. In particular, malvidin 3-glucoside, one of the main and more stable anthocyanins towards digestion, has been found in plasma and urine of human volunteers after red grape juice ingestion, indicating that it is not significantly degraded and is absorbed in its native (glycosylated) form [51]. Although there is controversy about the bioavailability of anthocyanins, it is now generally accepted that anthocyanin monoglycosides can be directly and rapidly absorbed and distributed in plasma at very low concentrations [47]. This particularity seems to be a key difference between the absorption of anthocyanins and other flavonoids [52]. 

Under our experimental conditions, the phenolic acids protocatechuic acid, caffeoyl quinic acid, fertaric acid, vanillic acid, ferulic acid and ellagic acid increased in the DIG compared with the ARE. This contrasts with other flavonoid compounds, such as resveratrol, myricetin and quercetin derivatives. The depletion of many native anthocyanins and the increase of phenolic acids and of other flavonoids in the DIG are intimately related and can possibly be explained by the ring cleavage of anthocyanins, followed by rearrangement and recombination of low molecular weight compounds to produce new polyphenols. 

For example, the significant increase of protocatechuic acid observed in the DIG is in complete agreement with what has been extensively described in the literature, since this compound corresponds to the major human metabolite of cyanidin 3-glucoside in humans [53]. It is thought that protocatechuic acid can be formed upon chemical degradation in the intestinal mucosa and in the systemic circulation [53]. Additionally, the increase in vanillic acid, another phenolic acid, in the DIG is in agreement with what happens in vivo. Nurmi et al. have reported that vanillic acid was one of the most abundant berry anthocyanin metabolites detected in humans [54]. 

The chemical changes observed in the DIG should be taken into consideration in their potential in the neuroinflammatory process. 

The aim of the second part of this work was to evaluate and compare the ability of the ARE with the DIG to reduce neuroinflammation in an LPS- and IFN-γ-stimulated microglial cell line. For this, a non-cytotoxic concentration for each of the extracts, 10 µg/mL of the total phenols, was selected.

According to the literature, signals, such as LPS and IFN-γ, can activate microglia cells, inducing their M1 phenotype. This leads to a pro-inflammatory response characterised, for example, by the upregulation of pro-inflammatory enzymes, specifically the iNOS and COX-2, which results in the overproduction of NO and PGE_2_, respectively. There is also upregulation of the secretion of inflammatory cytokines, such as TNF-α, or the overproduction of reactive oxygen species (ROS) [16,55,56,57]. This response can be detrimental to the brain and is closely related to neurotoxicity, and subsequently, to the development of diseases affecting the central nervous system [16]. 

In the present work, a mixture of LPS and IFN-γ was used to activate N9 cells. It was possible to observe that in LPS- and IFN-γ-exposed cells, the original anthocyanin-rich extract was able to efficiently inhibit either the production of NO (counteracting the mRNA expression of iNOS) or the secretion of PGE2, despite that the inhibition of mRNA COX-2 production did not show to be statistically significant

It is important to note that the DIG also inhibited, significantly, NO and PGE2 production, via downregulating iNOS and COX-2 mRNA expressions, in activated N9 cells. In the case of mRNA iNOS expression and NO production, the DIG provided even better protection than the non-digested extract, which indicates that the simulated digestion and, consequently, the final mixture of compounds produced during this process was more efficient in counteracting NO production than the original anthocyanin mixture, in activated microglial cells. It is well established that overproduction of NO, derived from iNOS overactivation, can be extremely noxious to the brain because it is related to the overproduction of reactive nitrogen species, such as the powerful peroxynitrite anion, which can cause serious cellular damage. This compromises the cellular integrity and cell viability, leading to DNA fragmentation and to mitochondrial dysfunction, contributing to the development of pathological conditions, such as Alzheimer’s and Parkinson’s diseases [58,59]. Thus, the protection afforded by both extracts against microglial cell activation is of extreme importance in the context of the combat against neuroinflammation-related brain disorders. 

It is also worth noting that both the ARE and the DIG reduced, significantly, under our experimental conditions, the levels of the well-known inflammatory cytokine TNF-α in LPS- and IFN-γ-stimulated cells. Interestingly with regard to this parameter, the simulated digestion did not impact negatively the anti-inflammatory efficiency of the polyphenolic extract. The TNF-α results complemented previous studies, reinforcing the anti-inflammatory role of both polyphenolic fractions. 

Knowing that overproduction of ROS occurs in neuroinflammation, and that this is strongly related to the development of various neurological disorders, through damage to neurons and creation of a vicious cycle of microglial activation and neuron damage [60], and considering that GSH depletion can also enhance the susceptibility of the brain cells to oxidative and inflammatory damage [61], it seemed pertinent to evaluate the antioxidant potential of the anthocyanin-rich extract, before and after in vitro digestion. This was studied in relation to their ability to reduce or increase ROS and GSH levels, respectively. 

In patients suffering from Parkinson’s disease, for example, a strong reduction in GSH levels has been reported in the *substantia nigra pars compacta* from post-mortem tissues. Thus, compounds able to increase GSH levels can be neuroprotective [61].

Under our experimental conditions, both polyphenolic fractions strongly reduced ROS levels and increased the production of the reduced form of glutathione (GSH), by promoting the mRNA expression of the enzyme GCL, for both catalytic and modifier subunits, in activated microglial cells. These results reaffirm the potential benefit of these natural compounds as preventive agents of neurological disorders, by combating neuroinflammation and oxidative stress. 

Since this work is intended to scrutinise the potential of anthocyanins extracted from Portuguese blueberries to combat microglia-driven neuroinflammation in a mechanistic perspective, the ability of the ARE and of the DIG to inhibit NF-kB cell signalling pathway was also ascertained. NF-kB is a transcription factor (p65/p50 is the best-characterised dimer) that when activated by signals, such as LPS, can freely translocate from the cytoplasm to the nucleus of the cell. After DNA binding, this induces the expression of multiple inflammatory genes, such as iNOS, COX-2 and TNF-α. NF-kB is considered a master regulator of M1 phenotype in microglia cells [60] and its downregulation can be advantageous to combat neuroinflammation. 

In this study, both the ARE and DIG were shown to be able to prevent the activation of NF-kB pathway, in LPS- and IFN-γ-exposed cells. However, since the DIG was shown to reduce the NF-kB activation even more efficiently than the non-digested extract, it is suggested that despite the changes that occur in the anthocyanin composition during the simulated digestion, the bioactivity of the original anthocyanin extract towards NF-kB suppression seemed to be improved, and thus, can be considered a promising strategy against neuroinflammation. Such involvement of the NF-kB pathway in the protection offered by polyphenolic compounds to counteract neuroinflammation has been corroborated by other authors [42,62,63]. 

Furthermore, as well as NF-kB, involvement of the JAK/STAT1 cascade in the anti-inflammatory and antioxidant protection afforded by both extracts was also evaluated. In general, the binding of IFN-γ to its receptor leads to the phosphorylation of JAKs and subsequently to the phosphorylation of STAT1 on its tyrosine (Tyr) residue, at position 701, making it ready to homodimerise and translocate to the cell nucleus. There, it binds to DNA and induces the transcription of genes, such as those encoding inflammatory enzymes, e.g., iNOS. 

Under our experimental conditions, the inflammatory stimulus, the combination of LPS and IFN-γ, did not affect the activation of STAT1 in microglial cells, suggesting that the protection provided by both extracts was related to STAT1-independent mechanisms. In fact, although STAT1 is the main pathway to transcription activated by IFN-γ, this cytokine can activate other transcription factors, in particular NF-kB, in a STAT1-independent process [64]. These mechanisms have not been fully clarified yet and require further research. 

## 5. Conclusions

In conclusion, the present work provides new insights on the chemical changes observed in an anthocyanin-rich extract obtained from Portuguese blueberries after simulated digestion, and about the potential of both the ARE and DIG to combat microglia-driven neuroinflammation. This work showed, for the first time, that this anthocyanin extract, after subjecting to an in vitro digestion, maintains its ability to reduce NO and PGE2 levels by reducing the mRNA production of iNOS and COX-2, respectively, in activated microglial cells, and is even more efficient than the original anthocyanin mixture. Furthermore, this digested fraction was also able to significantly reduce the ROS production and to increase the GSH levels in these cells. These protective effects seemed to be related to the reduction of the activation of NF-kB pathway and to STAT1-independent mechanisms.

It is important to highlight that the literature supports the ability of many anthocyanins to modulate brain inflammatory responses after crossing the blood-brain-barrier (BBB). For example, in vivo studies confirmed the ability of malvidin and cyanidin derivatives to cross the BBB and target the brain in their intact forms [65,66]. Moreover, in a very recent remarkable study by Grabska-Kobylecka et al., it was reported that phenolic acids, such as caffeic acid, homovanillic acid and 3-hydroxyphenyl acetic acid, were present in human cerebrospinal fluid samples [67]. Knowing that the only source of caffeic acid in human body is food, this suggests that this phenolic acid can cross the BBB in humans, paving the way for the possibility for other phenolic acids, such as those obtained in the present study, to be transported across the BBB and target the brain.

Based on the obtained data and taking into consideration the potential of anthocyanins and phenolic acids to cross the BBB, the anthocyanin-rich extract derived from Portuguese blueberries can be considered an interesting potential strategy to combat the neuroinflammatory process, which may be potentially useful in the future for the prevention of neuroinflammation-related neurological disorders, such as Parkinson’s and Alzheimer’s diseases. 

## Figures and Tables

**Table 1:** Compounds putatively identified by HPLC-DAD-MS/MS in the original blueberry extract (ARE) and in the digested fraction (DIG) and the corresponding recoveries after digestion. RT: Retention time. Major compounds are presented in bold, as presented in Figure 1. The recovery was calculated comparing the peak areas of the respective extracted-ion chromatograms of both extracts.

Peak	RT (min)	λmax	m/z ([M+H]^+^)	MS/MS	Putative ID	Original Extract (ARE)	Digested Extract (DIG)	Recoveryears (%)	References
**1**	**19.15**	-	627	303; 285; 464; 333	Delphinidin 3, 5-*O-*diglucoside	ARE	-	-	[31]
2	20.00	-	597	303	Delphinidin 3-*O*-sambubioside	ARE	-	-	[32]
**3**	**20.46**	**522**	**465**	**303; 257; 229; 153; 150**	**Delphinidin 3-*O*-galactoside**	**ARE**	**-**	**-**	[13,33]
4	19.79	285	167 (-)	152; 108; 36; 123	Vanillic acid	ARE	DIG	**83**	[34]
5	20.74	279	205	118; 146; 144; 132	Tryptophan	ARE	DIG	**779**	[35]
6	20.76	280	315 (-)	153; 123	Protocatechuic acid 4-*O*-glucoside	ARE	DIG	**136**	[36]
7	21.03	287, 322	355 (-)	192; 193	Ferulic acid hexoside I	ARE	DIG	**473**	[36]
8	21.16	-	515 (-)	191	Dicaffeoylquinic acid I	ARE	DIG	**184**	[34]
**9**	**21.20**	**522**	**465**	**303; 229; 257; 153**	**Delphinidin 3-*O*-glucoside**	**ARE**	**-**	**-**	[13,33]
**10**	**22.35**	**522**	**449**	**287; 137; 213; 241; 231; 269**	**Cyanidin 3-galactoside**	**ARE**	**DIG**	**8**	[13,33]
11	22.50	284, 321	433 (-)	300; 301	Ellagic acid pentoside	ARE		-	[35]
12	22.87	287, 318	355 (-)	192; 193	Ferulic acid hexoside II	-	DIG	-	[36]
13	23.85	517	449	287; 137; 213; 241; 231; 269	Cyanidin 3-glucoside	ARE	DIG	**2**	[13,33]
14	24.12	263, 324	325 (-)	192; 193; 165	Fertaric acid	-	DIG	-	[36]
**15**	**24.29**	**525**	**479**	**317; 302; 274; 257; 217; 203**	**Petunidin 3-galactoside**	**ARE**	**DIG**	**3**	[13,33]
16	24.49	280	227 (-)	135; 153	Resveratrol	-	DIG	-	[36]
17	25.40	522	595	287; 331; 247	Cyanidin 3-rutinoside	ARE	-	-	[35]
18	25.70	522	419	286; 109; 149; 129	Cyanidin 3-arabinoside	ARE	DIG	3	[13,33]
19	25.90	524	705 (-)	513	Delphinidin hexoside dimmer I	ARE	DIG	**110**	-
**20**	**25.99**	**522**	**479**	**317; 302; 274; 203; 85; 245**	**Petunidin 3-glucoside**	**ARE**	**DIG**	**3**	[13,33]
21	26.49	-	515 (-)	191	Dicaffeoylquinic acid II	ARE	DIG	**111**	[34]
**22**	**27.64**	**289, 315**	**355 (-)**	**193; 134; 149**	**Ferulic acid hexoside III**	**ARE**	**DIG**	**138**	[36]
23	28.06	526	463	301; 286; 203; 258	Peonidin 3-galactoside	ARE	DIG	17	[13,33]
**24**	**28.29**	**526**	**449**	**317; 302; 274; 245**	**Petunidin 3-arabinoside**	**ARE**	**-**	**-**	[13,33]
25	29.60	-	515 (-)	191	Dicaffeoylquinic acid III	ARE	DIG	**121**	[34]
**26**	**30.49**	**525**	**493**	**331; 315; 287; 270; 299; 150**	**Malvidin 3-galactoside**	**ARE**	**DIG**	**19**	[13,33]
27	30.54	525	463	301; 286; 213; 258	Peonidin 3-glucoside	ARE	DIG	**17**	[13]
28	32.67	326, 295	353 (-)	191; 85	Caffeoyl quinic acid	ARE	DIG	**186**	[36]
**29**	**33.13**	**525**	**493**	**331; 315; 287; 270; 299; 242; 179**	**Malvidin 3-glucoside**	**ARE**	**DIG**	**18**	[13,33]
**30**	**36.59**	**526**	**463**	**331; 315; 287; 270; 179; 150**	**Malvidin-3-arabinoside**	**ARE**	**DIG**	**14**	[13,33]
31	39.35	525	433	85; 86; 72; 301; 124; 182	Peonidin 3-arabinoside	ARE	DIG	**85**	[13]
**32**	**51.25**	**522**	**705 (-)**	**513; 339; 300**	**Delphinidin hexoside dimmer II**	**ARE**	**DIG**	**21**	-
**33**	**53.69**	**517**	**705 (-)**	**513**	**Delphinidin hexoside dimmer III**	**ARE**	**DIG**	**21**	-
34	55.35	524	535	331; 315; 287; 299; 270; 242	Malvidin 3′-(6″-acetyl-galactoside)	ARE	DIG	**12**	[33]
35	58.33	347, 525	319	153; 165; 111; 273; 245; 301	Myricetin	ARE	DIG	**35**	[34]
36	60.64	-	475 (-)	-	Ellagic acid 4-acetylpentoside	ARE	DIG	**146**	[35]
**37**	**65.42**	**529**	**535**	**331; 315; 287; 299; 179; 270; 242**	**Malvidin 3′-(6″-acetyl-glucoside)**	**ARE**	**DIG**	**11**	[33]
38	68.27	-	611	303; 166; 71; 238; 350; 137; 153	Rutin	ARE	DIG	**67**	-
**39**	**69.08**	**348**	**611**	**303; 129; 85; 71; 145; 137; 153; 229**	**Hesperidin**	**ARE**	**DIG**	**80**	-
40	68.32	-	465	303; 85; 137; 153; 229; 257; 165	Quercetin hexoside I	ARE	DIG	**72**	[33]
41	68.99	354	465	303; 85; 137; 153; 229; 257; 165	Quercetin hexoside II	ARE	DIG	**88**	[33]
42	69.86	-	465	303; 85; 137; 153; 229; 257; 165	Quercetin hexoside III	ARE	DIG	**35**	[33]
43	72.00	272	477 (-)	301	Quercetin 3-glucuronide	ARE	DIG	**49**	[35]
44	72.76	-	287	121; 241; 145; 153	Cyanidin	ARE	DIG	**71**	[34]
45	72.96	348	625	317; 302; 153; 139; 285; 274	Isorhamnetin-3-O-rutinoside/Isorhamnetin-3-O-galactoside-6”-rhamnoside/Myricetin 3-O-rutinoside	ARE	DIG	**79**	-
46	73.37	353	625	317; 85; 129; 243; 75; 111; 302; 285; 153; 274	Isorhamnetin-3-O-rutinoside/Isorhamnetin-3-O-galactoside-6″-rhamnoside/Myricetin 3-O-rutinoside	ARE	DIG	**83**	-
47	73.78	350	549 (-)	505; 300; 301; 355; 429; 63	Quercetin 3-O-(6″-malonyl-glucoside)	ARE	DIG	**42**	[36]
48	74.52	-	509	347; 103; 314; 85; 287; 286; 286; 153; 331; 139	Syringetin-3-O-galactoside	ARE	-	-	-
49	75.72	-	509	347; 287; 229; 291; 165; 286; 153; 331; 139	Syringetin-3-O-glucoside	ARE	-	-	[33]

## Supplementary Materials

The following are available online at https://www.mdpi.com/2072-6643/12/12/3670/s1, Figure S1: Effects of the original extract of Portuguese blueberries (ARE) and of the digested fraction (DIG) on cell viability of N9 cells. Cells were pre-incubated with 2.5, 5 and 10 µg/mL ARE or 2.5, 5 and 10 µg/mL DIG for 24 h. Cell viability was assessed by MTT test as described in “Materials and Methods and determined as percentage of control cellss. Values are mean ± SEM of at least three different experiments, in duplicate. * *p* < 0.05 vs. Control.
