# Supplementary material for: An Anthocyanin-Rich Extract Obtained from Portuguese Blueberries Maintains Its Efficacy in Reducing Microglia-Driven Neuroinflammation after Simulated Digestion
Source: Nutrients. 2020 Nov 28;12(12):3670. doi: 10.3390/nu12123670 (PMC7761390; doi:10.3390/nu12123670)
Supplement: Supplementary file 1 [file nutrients-12-03670-s001.pdf]

Article

An Anthocyanin-Rich Extract Obtained from Portuguese Blueberries Maintains Its Efficacy in Reducing Microglia-Driven Neuroinflammation after Simulated Digestion

Diana Serra [1,2,*], Joana F. Henriques [1,2], Teresa Serra [3,4], Andreia Bento Silva [5], Maria Rosário Bronze [4,5], Teresa C. P. Dinis [1,2] and Leonor M. Almeida [1,2]

[1] CNC—Center for Neuroscience and Cell Biology, 3000-504 Coimbra, Portugal; dianajserra@gmail.com (D.S.); joanafhenriques@gmail.com (J.F.H.); tcpdinis@ci.uc.pt (T.C.P.D.); malmeida@ci.uc.pt (L.M.A.)

[2] Faculdade de Farmácia, Universidade de Coimbra, 3000-548 Coimbra, Portugal dianajserra@gmail.com (D.S.); joanafhenriques@gmail.com (J.F.H.); tcpdinis@ci.uc.pt (T.C.P.D.); malmeida@ci.uc.pt (L.M.A.)

[3] IBET—Instituto de Biologia Experimental e Tecnológica, 2780-157 Oeiras, Portugal; tserra@ibet.com (T.S.)

[4] ITQB—Instituto de Tecnologia Quimica e Biologica Antonio Xavier, Universidade Nova de Lisboa, 2780-157 Oeiras, Portugal; tserra@ibet.com (T.S.); mbronze@ibet.pt (M.R.B.)

[5] Faculdade de Farmácia, Universidade de Lisboa, 1649-003 Lisboa, Portugal; abentosilva@ff.ulisboa.pt (A.B.S.); mbronze@ibet.pt (M.R.B.)

* Correspondence: dianajserra@gmail.com (D.S); Tel.: +351-239-488-400; Fax: +351-239-488-503

Supplementary information:

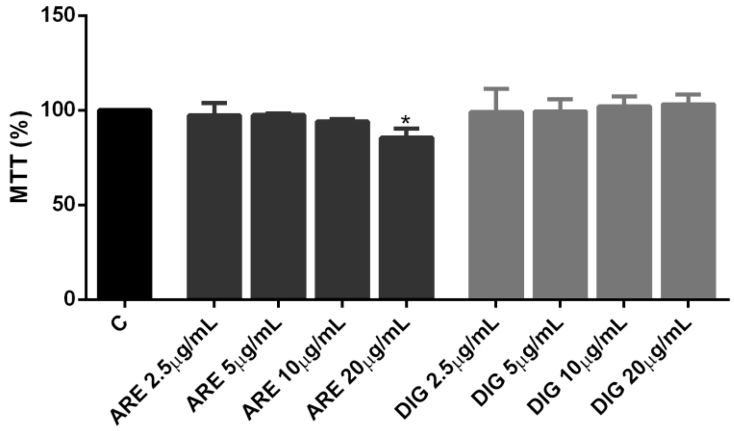

Supplementary figure. Effects of the original extract of Portuguese blueberries (ARE) and of the digested fraction (DIG) on cell viability of N9 cells. Cells were pre-incubated with 2.5, 5 and 10 μg/mL ARE or 2.5, 5 and 10 μg/mL DIG for 24 hours. Cell viability was assessed by MTT test as described in "Materials and Methods and determined as percentage of control cells. Values are mean ± SEM of at least three different experiments, in duplicate. **p* < 0.05 vs Control.
